# Situation analysis of procurement and production of multiple micronutrient supplements in 12 lower and upper middle‐income countries

**DOI:** 10.1111/mcn.12500

**Published:** 2017-12-26

**Authors:** Eva C. Monterrosa, Kalpana Beesabathuni, Kesso G. van Zutphen, Georg Steiger, Roland Kupka, Alison Fleet, Klaus Kraemer

**Affiliations:** ^1^ Sight and Life Basel Switzerland; ^2^ Independent Consultant Geneva Switzerland; ^3^ Nutrition Improvement Program, DSM Kaiseraugst Switzerland; ^4^ UNICEF Programme Division New York New York USA; ^5^ UNICEF Supply Division Copenhagen Denmark; ^6^ Johns Hopkins School of Public Health Baltimore Maryland USA

**Keywords:** affordability, manufacturing capacity, multiple micronutrient supplements, policy, regulations, trade agreements

## Abstract

Globally, there are few vitamin and mineral ingredient manufacturers. To support local, in‐country or regional procurement and production of multiple micronutrient supplements (MMS), the following production scenarios are possible: (a) straight ingredients of vitamins and minerals forms imported or locally produced that are mixed, tableted, or encapsulated and packaged by a local manufacturer; (b) import or local production of a vitamin and minerals premix that is tableted or encapsulated and packaged locally; (c) import of a bulk, finished product (tablets or capsules) that is packaged and branded; and (d) or import of a branded packaged product. This paper is a situation analysis of the market, manufacturing, and policy factors that are driving the production of MMS in 12 lower and upper middle‐income countries. Key informants completed a self‐administered structured questionnaire, which examined the local context of products available in the market and their cost, regulations and policies, in Brazil, Colombia, Guatemala, Mexico, Peru, Bangladesh, India, Vietnam, Ghana, Kenya, Nigeria, and South Africa. Our study found that although most countries have the capacity to produce locally MMS, the major barriers observed for sustainable and affordable production include (a) poor technical capacity and policies for ensuring quality along the value chain and (b) lack of policy coherence to incentivize local production and lower the manufacture and retail price of MMS. Also, better guidelines and government oversight will be required because not one country had an MMS formulation that matched the globally recommended formulation of the United Nations Multiple Micronutrient Preparation (UNIMMAP).

## 1 INTRODUCTION

Maternal undernutrition during pregnancy continues to be a major global health problem (Black et al., 2013). Mothers who are undernourished during pregnancy are at greater risk for preterm delivery and for delivering infants who are small for gestational age or at low birth weight (Abu‐Saad & Fraser, 2010). Breaking the intergenerational cycle of malnutrition therefore requires addressing undernutrition during gestation. Available evidence suggests that multiple micronutrient supplements (MMS) during pregnancy reduce the risk of low birthweight and small for gestational age compared to mothers who received iron‐folic acid (IFA) only (Haider & Bhutta, 2017). Compared to women receiving iron alone, with or without folic acid, women receiving MMS with iron and folic acid had about a 10% reduction in risk of delivering small‐for‐gestational age infants (RR 0.92, 95% CI [0.83, 0.97]; 14 trials), and low‐birthweight infants (RR 0.88, 95% CI [0.85, 0.91; 15 trials]. Although the current evidence is not sufficient for World Health Organization (WHO) to issue a global recommendation, WHO did recommend in their antenatal guidelines that “policymakers in populations with a high prevalence of nutritional deficiencies might consider the positive effects of multiple micronutrient supplement on maternal health to outweigh the disadvantages, and might choose to give multiple micronutrient supplement which include IFA” (WHO, 2016).

As policy leaders weigh the health benefits of MMS during pregnancy, programming for large‐scale micronutrient interventions, such as MMS, must address several upstream processes for delivery and scale up (Menon et al., 2014); considerations related to the local procurement of MMS are crucial to guarantee a stable, affordable, and high‐quality supply of MMS (Bhutta, Imdad, Ramakrishnan, & Martorell, 2012; Shrimpton, Huffman, Zehner, Darnton‐Hill, & Dalmiya, 2009). The supply of MMS in country will be influenced not only by local manufacturing capacities but also by the regulatory policies that enable manufacturing scenarios, as well as the capacity of countries to monitor the quality of MMS along the value chain.

Globally, there are few vitamin and mineral ingredient manufacturers. For in‐country or regional procurement of MMS, there are four possible scenarios (Table 1). Scenarios A and B require local manufacturing capacities for blending and mixing; scenario C is likely to be found in countries with lower manufacturing capabilities; and scenario D in countries with no capacity or with small consumer markets.

**Table 1:** Description of the four scenarios likely to be encountered in lower and upper middle‐income countries for procurement and production of multiple micronutrient supplements

Scenario A—straight ingredients^a^	Scenario B—premix	Scenario C—bulk finished products	Scenario D—international branded product
Import or local production of pure vitamins or mineral forms, in liquid or dry form, that are subsequently blended, tableted or encapsulated and packaged, distributed, and marketed	Import or local production of premix blend of vitamins and minerals or other nutrition ingredients that are tableted or encapsulated and packaged, distributed, and marketed	Import of bulk finished product that is packaged, distributed, and marketed as international or local brand	Finished, branded product is imported, distributed, and marketed

a The term straight ingredients is preferred as it includes both active pharmaceutical ingredient (vitamin) and its forms for dry tableting.

This paper explores these four scenarios by examining the manufacturing capacities and policies for MMS production that exist in a sample of 12 lower and upper middle‐income countries (hereafter middle‐income countries, MIC).

**Key messages**

- Despite the availability of MMS in the marketplace, not one survey country has a product that matches the internationally recommended UNIMMAP formulation.
- In some countries where imported brands are preferred by consumers, local companies will need to invest in marketing to compete with international brands.
- Regional trade agreements or technology transfer should be leveraged to ensure affordable local supply of high‐quality MMS.
- Monitoring and surveillance systems need to be strengthened, and local food and drug authorities need better oversight on the MMS quality and potency.
- To achieve policy coherence, national governments may need to consider consumer‐facing and manufacturing‐friendly policies to ensure affordable supply of MMS.

## 2 METHODS

We selected 12 MIC and 3 high‐income countries (HIC) that were included for each region as a comparison. MIC were selected if they met two criteria: (a) presence of DSM staff to facilitate data gathering, and (b) countries with moderate to poor maternal health indicators during pregnancy as indicated by low birthweight or maternal anaemia in pregnancy (Table 2). We selected the following countries: Brazil, Colombia, Guatemala, Mexico, Peru, (for Latin America [LATAM]), Bangladesh, India, Vietnam (for Asia), Ghana, Kenya, Nigeria, and South Africa (for sub‐Saharan Africa). Because of the strong manufacturing and quality control (QC) policies for MMS in the United States, Japan, and Germany, and their potential as regionally suppliers for MMS, these countries served as comparison for LATAM, Asia, and Africa, respectively (Wallace, 2015).

**Table 2:** Prevalence of anaemia among pregnant women ages 15–49 years and prevalence of children under 5 years affected by low birth weight

	Prevalence of pregnant women aged 15–49 years with anaemia (%)^a^	Prevalence of children under 5 affected by low birth weight (%)^b^
Latin America		
Brazil	32	8
Colombia	30	6
Guatemala	30	11
Mexico	21	9
Peru	23	8
Asia		
Bangladesh	48	22
India	54	28
Vietnam	24	5
Sub‐Saharan Africa		
Ghana	62	11
Kenya	36	8
Nigeria	58	15
South Africa	30	N/A
Regional Aggregates		
East Asia and Pacific	39	6
Latin American & Caribbean	28	9
South Asia	39	28
Sub‐Saharan Africa	44	13

a Source: WHO 2015 (WHO, 2015). Data ranged from 1995 to 2011. Anaemia is defined as blood haemoglobin concentration <110 g/L for pregnant women. Regional aggregates include all countries in the region

b Source: UNICEF Statistics (UNICEF, 2016). Data ranged from 2005 to 2012. Low birth weight is a weight at birth of less than 2,500 g irrespective of gestational age. N/A: data not available.

**Figure 1:**
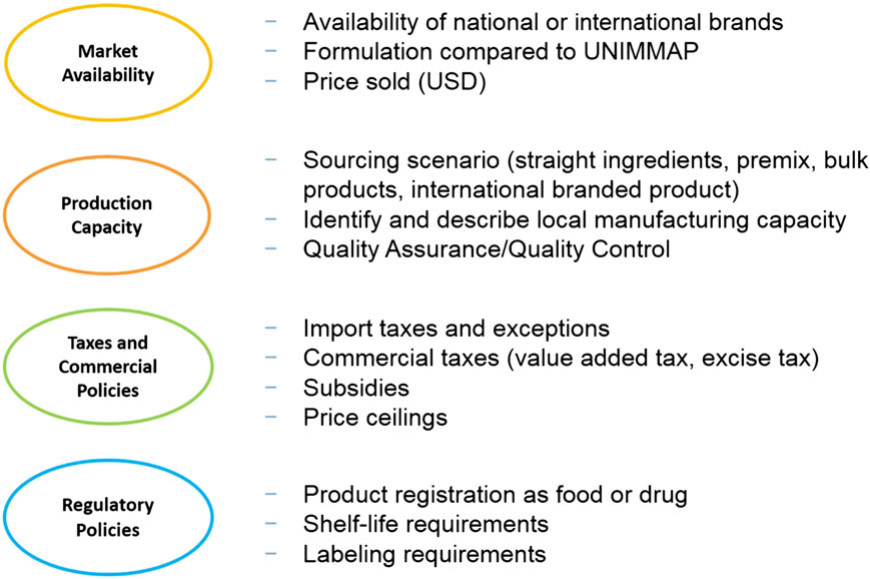
Conceptual framework identifies four domains describing the conditions for local procurement and production of multiple micronutrient supplement within a given country

A 40‐question, self‐administered questionnaire was designed to collect data on four key areas: product availability in local markets, production capacity, taxes and commercial policies, and regulatory landscape (Figure 1). The questionnaire was sent to key informants (employees or key stakeholders) of DSM with knowledge about the local consumer market and regulatory policies. Data on policies and regulations were augmented by key informants in the regulatory affairs unit of DSM with knowledge in these markets. Information from this questionnaire was verified with each key informant and, where possible, cross‐checked via web search or with information available to the United Nations Children Fund (UNICEF).

The data collection was restricted to supplements containing vitamins and minerals in tablets or capsules for adults (either targeted to male or female needs) or for women who were pregnant or lactating. We excluded supplements for children, products sold as syrups and powders, due to poor stability and palatability of these products, and those that contained herbal ingredients for lack of scientific evidence in using these products for improved pregnancy outcomes. For countries with high manufacturing capabilities and numerous MMS brands on the market, we restricted the analysis to the top 5 selling brands/companies.

For these countries, we assumed that local production or availability of MMS could be achieved via four scenarios as described in Table 1. In situations where these scenarios did not apply, we inquired about MMS production locally, and policy and market factors.

Pictures of products and product labels were collected in each of the 12 MIC. Only product label information was analysed. The UNICEF/WHO/United Nations University (UNU) international multiple micronutrient preparation (UNIMMAP) formulation was considered the benchmark for ingredients and recommended daily dose due to the scientific evidence of its efficacy on pregnancy outcomes (UNICEF, WHO, and UNU, 1999; West et al., 2015). Beta‐carotene doses were converted to vitamin A in μg for comparison. Four nonoverlapping categories between the product and UNIMMAP were created: (I) less than 10 ingredients and dosage consistent with UNIMMAP; (II) at least 10 ingredients and dosage consistent (80% or more of the recommended dosage) with UNIMMAP; (III) ingredients and dosage consistent with UNIMMAP (i.e., with 80% or more of the recommended dosage); and (IV) ingredients and dosage consistent with UNIMMAP and have other key nutrients. Category III is the benchmark.

Key informants were asked to report on local manufacturing capacity either by identifying installed capacity (or rated capacity, i.e., maximum capacity that the system is designed to run at) or sales of MMS by top local manufactures. If information for installed capacity was not reported, as proxy we took sales of the local MMS brands through pharmacies, information available on the IMS Health database (Danbury, CT). We asked if facilities are WHO‐Good Manufacturing Practices (GMP) certified and if capital goods could be easily acquired for mass production of MMS. Key informants also reported major barriers faced for good quality assurance (QA) and quality control (QC) from sourcing ingredients, blending process, production of capsules or tablets, packaging, transport conditions, warehousing conditions, and storage conditions at point of purchase.

All local currencies were converted to U.S. dollars (USD), using the currency's 12‐month average conversion rate (June 2014 to May 2015). For numeric response items, we conducted simple descriptive statistics and reported mean (unweighted average) and standard deviations. Linear regression was done in STATA (College Station, TX) to assess product price differences for (a) low‐income countries versus middle‐income countries, (b) dose Category I versus dose Category II, and (c) local versus imported. For nominal and qualitative responses items, responses were summarized into tables by region and country to allow for descriptive comparisons.

## 3 RESULTS

### 3.1 Market availability of MMS

Variations in MMS categories and prices can be best described by region—LATAM, Africa, and Asia (Tables 3a and 3b). Seventy‐one products were collected from 12 MIC and 12 products from 3 HIC. These products belong to either Category I or II, as defined in Section 2. There were no products belonging to categories III or IV. Two MMS, one from Nigeria and the other from India, would have exactly matched with UNIMMAP formulation (and hence classified as Category III) if they had 40% more iron. Similarly, MMS specified for public sector channels by the government of South Africa would have exactly matched with UNIMMAP formulation if it had 38% more copper. Both local and imported MMS can be found across the regions. Of these 71 MMS, the desired products for antenatal supplements are those that are locally manufactured and belong to Category II. There are 15 such products. Among these, 10 are from Asia, 4 from LATAM, and 1 from South Africa. The mean retail price of these 10 MMS from Asia is one fourth of those found in LATAM. So local supplements from Asia seem to be more affordable than those from LATAM.

**Table 3a:** Selected variables and prices (in USD) for a package or a bottle of 30 MMS in Latin America, Africa, and Asia regions

	Countries		
Variables	Latin America^a^	Africa	Asia
	Brazil, Colombia, Guatemala, Mexico, Peru	Ghana, Kenya, Nigeria, South Africa	Bangladesh, India, Vietnam
Local	13.59 ± 6.65 (*n* = 7)	7.79 ± 4.94 (*n* = 5)	3.96 ± 2.58 (*n* = 16)
Imported	13.39 ± 6.85 (*n* = 13)	10.75 ± 6.70 (*n* = 27)	8.30 ± 2.89 (*n* = 2)
Category I	10.48 ± 5.34 (*n* = 7)	9.36 ± 7.85 (*n* = 12)	4.68 ± 2.81 (*n* = 8)
Category II	15.20 ± 6.82 (*n* = 13)	11.05 ± 6.28 (*n* = 21)	4.26 ± 3.09 (*n* = 10)
Category II + Local	16.26 ± 5.79 (*n* = 4)	4.26 (*n* = 1)	4.19 ± 2.94 (*n* = 10)
Total no. of local and imported products	20	33^b^	18

*Note*. MMS = multiple micronutrient supplement.

a Mean price (in USD)  ± SD (unweighted). Category I—less than 10 ingredients and dosage (80% or more of the recommended dosage) consistent with UNIMMAP; Category II—at least 10 ingredients and dosage consistent with UNIMMAP.

b Although the total number of products sampled in the Africa region was 33, for one product we were unable to verify if it was local or imported.

**Table 3b:** Selected variables and prices (in USD) per capsule or tablet of MMS in Latin America, Africa, and Asia regions

MMS	Latin America^a^	Africa	Asia
Variables	Brazil, Colombia, Guatemala, Mexico, Peru	Ghana, Kenya, Nigeria, South Africa	Bangladesh, India, Vietnam
Local	0.453 ± 0.222 (*n* = 7)	0.260 ± 0.165 (*n* = 5)	0.132 ± 0.086 (*n* = 16)
Imported	0.446 ± 0.228 (*n* = 13)	0.371 ± 0.228 (*n* = 27)	0.277 ± 0.097 (*n* = 2)
Category I	0.349 ± 0.178 (*n* = 7)	0.312 ± 0.262 (*n* = 12)	0.156 ± 0.094 (*n* = 8)
Category II	0.480 ± 0.223 (*n* = 13)	0.375 ± 0.206 (*n* = 21)	0.142 ± 0.103 (*n* = 10)
Category II + Local	0.542 ± 0.193 (*n* = 4)	0.142 (*n* = 1)	0.140 ± 0.103 (*n* = 10)
Total no. of local and imported products	20	33^b^	18

*Note*. MMS = multiple micronutrient supplement.

a Mean price (in USD)  ± SD (unweighted). Category I—less than 10 ingredients and dosage consistent (80% or more of the recommended dosage) with UNIMMAP; Category II—at least 10 ingredients and dosage consistent with UNIMMAP.

b Although the total number of products sampled in the Africa region was 33, for one product we were unable to verify if it was local or imported.

With the exception of South Africa, none of the MIC make MMS available through government channels. Among the three HIC surveyed (i.e., Germany, Japan, and the United States), MMS was available through government channels only in the United States. A detailed description of results by region follows.

#### 3.1.1 Latin America

Both local and imported MMS in blister or bottle packaging are found in the five LATAM countries surveyed—Brazil, Colombia, Guatemala, Mexico, and Peru. MMS can be bought in pharmacies in all the five countries. It is also available through pharmacy websites (e‐commerce) in Brazil and Colombia. In Colombia, Guatemala, Mexico, and Peru, MMS can also be bought in supermarkets. Direct selling or home delivery of MMS can be found in Guatemala, Mexico, and Peru. MMS for pregnancy is not available through government institutions in all of the five countries. In Guatemala, MMS for pregnancy is available at antenatal care with a private doctor. So MMS can be found through one or more commercial channels in each of these five countries.

Twenty MMS brands available in LATAM were compared for this study. In all, 35% (*n* = 7) are manufactured locally, and 65% (n = 13) of the MMS sampled fall into Category II, where at least 10 ingredients and dosage are consistent with UNIMMAP. The remaining supplements belong to Category I. The Category II supplements lack significant amounts of one or more of vitamin B3 (niacin) and minerals such as copper and selenium (0% to 50% of the recommended dosage). Mean retail price for a pack of 30 tablets or capsules are reported in Table 3a.

MMS in Category II found in Colombia and Mexico also contain one or more of other key nutrients such as biotin, calcium, vitamin K, magnesium, potassium, and DHA (docosahexaenoic acid, an omega 3 fatty acid) at the same or slightly higher prices compared to other supplements in this category. This indicates that manufacturers in Colombia and Mexico may offer competitive and affordable MMS that matches the UNIMMAP formulation.

The average price for package of 30 tablets from Category II sold in the United States were comparable in price (USD 14.70 ± 1.39) to similar products sold in the five Latin American countries (USD 15.20 ± 6.82 for a pack of 30 capsules or tablets). However, the price of MMS from the United States varies considerably depending on the retail location. For example, in drugstores, wholesale stores and e‐commerce, the price per capsule or tablet is significantly lower (nearly 75%) than the range mentioned above. Also, these supplements, in both Categories I and II, in the United States lack significant amounts of one or more of vitamins A, B1, B2, and B3 (0% to 60% of the recommended UNIMMAP dosage).

#### 3.1.2 Africa

Both local and imported MMS in blister or bottle packaging are found in the four African countries surveyed—Ghana, Kenya, Nigeria, and South Africa. MMS can be purchased through several channels—pharmacy, supermarket, and “mom and pop” stores, and directly sold or home delivered to consumers in these four countries.

Thirty‐three MMS brands were compared in this study. Most of them (*n* = 27) are imported branded products. More than 60% of the products (*n* = 21) fall into Category II where at least 10 ingredients and dosage are consistent with UNIMMAP. The remaining (*n* = 12) supplements belong to Category I. The one imported product from Nigeria that nearly matches the UNIMMAP formulation retails for 20.50 USD for a package of 30 tablets. All of the MMS in Category II are imported except for one antenatal product manufactured in South Africa. This local MMS has an affordable retail price of USD 4.26 for a pack of 30 tablets. Further, when compared to the UNIMMAP formulation, this MMS brand manufactured in South Africa lacks 30% to 50% of the benchmark amount for three micronutrients—copper, selenium, and zinc—but contains other micronutrients such as vitamin K, biotin, pantothenic acid, and the essential amino acid L‐lysine. The remaining Category II supplements surveyed in this region lack significant amounts of one or more of vitamins A, D, E, minerals copper, iodine, iron, selenium, and zinc (0% to 60% of the recommended dosage), but they contain other essential micronutrients such as vitamin K, biotin, pantothenic acid, L‐lysine, fish oil, and DHA, which are not prescribed in the UNIMMAP formulation. Mean retail price for a pack of 30 tablets or capsules are reported in Table 3a.

MMS is available through government channels only in South Africa. The South African government issued tenders in 2011 and 2014 that include specifications of the MMS required for pregnant women. These MMS have been available for order by South African provinces that have a relevant policy in place. MMS are available in a container of 30 or 60 capsules and are consistent with UNIMMAP, except for dosage of copper (62% of the recommended dosage). In addition to 15 micronutrients from UNIMMAP, the MMS distributed through government channels have one other micronutrient, 55 to 75 μg of vitamin K.

#### 3.1.3 Asia

Both local and imported MMS in blister or bottle packaging are found in the three Asian MIC surveyed—Bangladesh, India, and Vietnam. In India and Japan, MMS can be purchased through several channels—pharmacy, supermarket, and “mom and pop” stores, or directly sold to consumers through online portals or home delivery. In Bangladesh, MMS is available only in pharmacies, whereas in Vietnam, MMS is available through a number of channels—pharmacies, mom and pop shops, private clinics, and direct selling through home shopping, which includes mail or telephone ordering from catalogues; telephone ordering in response to advertisements in print and electronic media (such as periodicals, TV, and radio) and online shopping. MMS for pregnancy is not available through government channels in any of these countries, except for Vietnam where it is available for purchase at hospitals. In Japan, MMS is not recommended by physicians to pregnant women unless micronutrient deficiencies are detected during a diagnosis.

Eighteen MMS brands from Bangladesh, India, and Vietnam were sampled in this study. Most of them are local products (*n* = 16). Nearly equal number of MMS belongs to Category I (*n* = 8) and Category II (*n* = 10). One MMS in Category II from India would have classified as Category III if it had 40% more iron. However, it contains other micronutrients such as biotin, pantothenic acid, and calcium. This is a locally made product available at a retail price of USD 9.77 for a pack of 30 tablets. The most affordable MMS in Category II is made in India, which is available in pharmacies at a retail price of USD 0.38 for a bottle of 30 tablets; however, this affordable MMS does not contain vitamin B3 (niacin), copper, selenium, and iodine.

MMS brands in Category II lacked significant amounts of one or more of vitamins B3, A, B6 and minerals iron, copper, selenium, zinc, and iodine (0% to 58% of recommended dosage) but include other key micronutrients for pregnancy, such as calcium and magnesium.

Mean retail prices for a pack of 30 supplements are reported in Table 3a. The four MMS from Japan belonged to Category I and retailed at USD 6.11 ± 6.23 for 30 tablets or capsules.

We also observed some general trends for pricing of MMS across regions (Tables 3a and 3b). In Africa and Asia, imported products tended to be more expensive than local products. This was not observed in LATAM, where imported products seemed to be competitively priced. However, the regression analyses show that in general, we can expect that imported products will be significantly more expensive by USD 4.25 per package of 30 tablets or capsules than local brands (Table 4). In LATAM and Africa, products were more expensive if they had more ingredients and higher dosage, though this difference was not statistically significant (Table 4). The regression analyses show that a package of 30 tablets or capsules was USD 4.23 lower in MIC than that in upper middle‐income countries (Brazil, Colombia, Mexico, Peru, and South Africa; Table 4).

**Table 4:** Factors associated with price (USD) per package of 30 capsule or tablet of multiple micronutrient supplement^a^

Variables	Coefficient (USD)	Std. Error	t	P > |t|	[95% CI]	
Lower middle income countries vs. Upper middle income*	−4.231561	1.56568	−2.70	0.009	−7.360322	−1.102799
Imported vs. Local*	4.255024	1.530501	2.78	0.007	1.196561	7.313487
Category II vs. I*	2.160423	1.567411	1.38	0.173	−.9717978	5.292644

a *N* = 71 products.

* Reference category. Upper middle‐income countries: Brazil, Colombia, Mexico, Peru, and South Africa. Lower middle‐income countries: Guatemala, Ghana, Kenya, Nigeria, Bangladesh, India, and Vietnam. Category I: less than 10 ingredients and dosage consistent (80% or more of the recommended dosage) with UNIMMAP. Category II: at least 10 or more ingredients and dosage consistent with UNIMMAP formulation.

### 3.2 Product registration

We ascertained whether MMS for adults or pregnancy were registered as food or drug in each country. Drugs undergo stringent controls for quality, and the cost of registration is high. Foods, on the other hand, are usually easier and cheaper to register in countries. The preference for drug over food registration might signal greater degree of government oversight/monitoring from production to market place. With regard to cost of registration, there were major differences between high‐income countries (HIC) and MIC, with Germany and Japan registration costing tens of thousands of dollars more than their regional analogues (Table 5).

**Table 5:** Registration cost for MMS supplement by category (food and/or drug) in Latin America, Africa, and Asia regions

	Registration cost (USD) for MMS supplement
Africa	
Ghana	As a food: 1,800
Kenya	As a drug: 2,000
Nigeria	As a food: 9,231
South Africa	As a food: 2,059
Germany^a^	As a food: 11,000
Americas	
Brazil	As a food: 567
	As a drug: 1,890
Colombia	As a food: 1,400
	As a drug: 1,320
Guatemala	As a food or drug: 500
Mexico	As a drug: 1,003
	As a food: no cost
Peru	As a drug: 10% of UIT (UIT in 2015: S/1210) = 121
United States^a^	Product registration with regulatory authorities is not required
Asia	
Bangladesh	As a food: 257
India	Either as food or drug: 6,293
Vietnam	Either as food or drug: 92 to 458
Japan^a^	Either as food or drug: 15,000

*Note*. MMS = multiple micronutrient supplement; UIT = Unidad Impositiva Tributaria.

a High‐income country used as comparator.

Table 5 also identifies the registration category for MMS by country. In Peru, MMS is registered as a drug, whereas in Brazil, Colombia, Guatemala, and Mexico, an MMS is registered as a drug only if it exceeds 100% of the local DRV standards. In Brazil, products registered as drugs were subject to price controls set by the regulatory agency. Kenya was the only country in Africa region to require drug registration for MMS. In India, registration as a food or drug depends entirely on the analysis of ingredients and the authorities, whereas in Vietnam, it depends on the percentage of DRV provided through the product.

### 3.3 Import taxes and exemptions

The import taxes reported by informants varied widely, with African countries having some of the highest taxes for each of the four scenarios (Table 6). Ghana, for example, reportedly charges a 38–40% tax for all nutritional products, whereas Peru imposes zero tax on straights or premix blends and a lower tax on imported, finished products. In some countries, the import tax varies by nutritional product. India applies a 7% tax to vitamins only, whereas minerals, vitamin‐mineral blends, and omega fatty acids are taxed at 30% of the import value. A similar, but less drastic scenario, is found in Guatemala, Mexico, and Colombia (Table 6).

**Table 6:** Taxes on sourcing of straights, premix blends, bulk capsules, branded/finished product in Latin America, Africa, and Asia regions

Sourcing Scenarios					
		A: Straights are imported	B: Premix are imported	C: Import of bulk finished product	D: Import international branded product
Standard Import taxes (% tax on value of import)	Africa				
	Ghana	38–40%			
	Kenya	16%	16%	16%	16%
	Nigeria	5%	10%	5%	20%
	South Africa	6–10% if from non‐EU	20% if from non‐EU	20% if from non‐EU	20% if from non‐EU
	Latin America				
	Brazil	0 to 16%	8%	8%	8%
	Colombia	0% Vitamins	0% Vitamins	0–5%	5–15%
		15% Omega‐3 fatty acids	5% Minerals		
	Guatemala	0%	0–15%	15%	15%
	Mexico	0–5%	0–15%	0–15%	0–15%
	Peru	0%	0%	6%	6%
	United States	Not applicable ‐ vitamin and minerals forms are manufactured locally			
	Asia				
	Bangladesh	5%	N/A	N/A	N/A
	India	7.5% vitamins	7.5% vitamins	7.5% vitamins	7.5% vitamins
		30% minerals & vitamin mineral blends	30% minerals & vitamin mineral blends	30% minerals & vitamin mineral blends	30% minerals & vitamin mineral blends
	Japan	0%	0%	0%	0%
	Vietnam	0–5%	0–5%	5–10%	5–10%
	Europe				
	Germany	—It depends on various criteria: the production facilities, what form they can produce, straights or premixes, economies of scale, etc.			

*Note.* N/A = data not available.

#### 3.3.1 Exemptions

Both Colombia and Mexico had exemptions to import taxes. Colombia does not levy taxes for 2 years on raw materials, which are not manufactured in country, meaning that straights and premixes would be tax exempt. Mexico, through NAFTA, offered import tax waivers for nutrition ingredients or products from the United States and Canada.

Of the Asian countries in our study, only Vietnam allowed exemptions for nutrition ingredients or products through various free trade agreements in the South Asia region (e.g., ATIGA, ASEAN Trade in Goods Agreement; ACFTA, ASEAN‐China Free Trade Agreement; AKFTA, ASEAN‐Korean Free Trade Agreement; AJCEP, ASEAN‐Japan Comprehensive Economic Partnership; VJEPA, Vietnam‐Japan Economic Partnership Agreement; and AANZFTA, ASEAN‐Australia‐New Zealand Free Trade Agreement).

In Africa, only South Africa offered import exemptions to nutritional ingredients from European Union (EU) countries via the EUR1 movement certificate.

### 3.4 Manufacturing scenarios and regional trade

#### 3.4.1 Preferred scenario

We asked key informants to discuss which scenario (Table 1) was preferred in their country, with the aim of understanding the factors that would encourage local manufacturing. In LATAM, straight scenario (Scenario A) is preferred across all four countries due to lower taxes and local manufacturing capacity for blending. Additionally, in Colombia, Mexico, and Peru, the premix scenario (Scenario B) is also preferred due to lower import taxes.

In Kenya, Ghana, and Nigeria, although some local manufacturing from straights and premix does take place, the preferred scenario is imports of bulk products (Scenarios C) or branded supplements (Scenario D) for several reasons identified by our key informants: (a) manufacturing capacity is low in these countries, (b) consumers prefer the branded supplements, and (c) there is a small consumer market for these products. In contrast, South Africa imports straights, premix, or bulk products, as the country has a higher manufacturing capacity, and takes advantage of free trade agreements with the EU. In the HIC Germany, the preferred scenario is to import or produce straights.

Asia, Japan, and Vietnam prefer to import straights or premix because of lower import taxes and high manufacturing capacity for blending. Bangladesh prefers to import straights.

India locally manufactures most vitamins (i.e., vitamin A, C, D, E, B2, B3, and B12, biotin, and folic acid) and manufactures straights for all minerals. When other vitamins are needed, the straight scenario would be used.

#### 3.4.2 Regional trade

We also explored whether trade agreements could facilitate regional production of MMS, if local production was not possible. Of the countries with lower manufacturing capacities, only Guatemala and Peru had regional trade agreements that would facilitate acquiring products produced regionally. In East Africa, regional MMS production may be facilitated with the common trade tariff among these countries.

### 3.5 Taxes and commercial policies—Incentives and disincentives

We also sought to understand how other economic policies might encourage (i.e., price subsidies and tax exemptions) or discourage (i.e., price ceilings) local manufacturing or may potentially limit consumer demand (i.e., commercial taxes). These are described below.

#### 3.5.1 Price subsidies

India was the only country with price subsidies for MMS, if those were manufactured in excise free zones. In the United States, branded supplements may be eligible for rebate structures through state programmes such as WIC (Special Supplemental Nutrition Program for Women, Infants, and Children). African countries did not have any price subsidies.

#### 3.5.2 Tax exemptions

Very few countries offer tax exemptions. Colombia offers exemptions for straights and premix blends of vitamins and on bulk products or branded supplements that are imported as drugs. In India, there are excise duty and sales tax exemptions for products manufactured in duty free zones. While in the United States, it is the state authorities that may grant sales tax exemptions to MMS products provided through social programmes.

#### 3.5.3 Commercial taxes

We also asked about commercial taxes because these are indirect taxes ultimately borne by the final consumer at point of purchase. Countries reported three types of commercial taxes: sales tax, value added tax, and excise tax. These commercial taxes are part of the retail price and are regressive policies because they place a greater economic burden on low‐income consumers compared to higher income groups. Excises are inland taxes typically imposed on producers and manufacturers and ultimately passed onto the consumer. The commercial taxes varied widely across the regions and within regions (Table 7) with some of the highest taxes paid on the imported, branded products. No data were available for Ghana or Japan.

**Table 7:** Commercial taxes for selected countries in Latin America, Africa, and Asia regions by sourcing scenario

		Sourcing Scenarios			
		A: Straights are imported or produced	B: Premix are imported or produced	C: Import of bulk finished product	D: Import international branded product
Commercial duties (% tax or amount in local currency)	Africa				
	Ghana	N/A	N/A	N/A	N/A
	Kenya	N/A	N/A	N/A	N/A
	Nigeria	None	None	None	None
	South Africa	14%	14%	14%	14%
	Latin America				
	Brazil	22%	38%	38%	38%
	Colombia	0% (Vitamins) 16% (Minerals & Omegas)	VAT: 0% (current situation), but local authority would like to apply value added tax of 16% on premix that includes more than vitamins	16% supplement 0% drug	16% supplement 0% drug
	Guatemala	0%	0–15%	12%	12%
	Mexico	0–16%	0%	16%	16%
	Peru	18%	18%	18%	18%
	Asia				
	Bangladesh	N/A	N/A	N/A	15%
	India	Excise at 12–50% + local sales tax at 12.5%	Excise at 12–50% + local sales tax at 12.5%	Excise at 12–50% + sales tax at 5.5%	Excise at 12–50% + sales tax at 5.5%
	Japan	N/A	N/A	N/A	N/A
	Vietnam	10% value added tax +28% company income tax	10% value added tax + 28% company income tax	10% value added tax+ 28% company income tax	10% value added tax + 28% company income tax

*Note.* N/A = data not available.

#### 3.5.4 Price ceilings

There was little use of price ceilings for MMS across the countries, except for Brazil and India. In Brazil, MMS is registered as drug so it needs to be submitted to the National Price Agency for Drugs. In India, prices for MMS are set by the National Pharmaceutical Pricing Authority.

### 3.6 Regulatory policies

#### 3.6.1 Shelf‐life requirements

Shelf‐life requirements are important for every stage of the production process to ensure the active ingredients, intermediates, and the finished product be delivered to the full potency. Procurement of ingredients, production process, and choice of packaging materials are governed by the shelf‐life requirements. For example, manufacturers, who are not willing to take a risk on their brand, source very high‐quality ingredients, with a longer shelf life. Laws governing shelf‐life requirements for imports were very diverse within and across regions (Table 8). In some countries, the shelf‐life requirements were mandatory (Peru, Ghana, Kenya, India, and Vietnam), but in other countries, only guidelines or limits were imposed by the importing manufacturing or retailer (South Africa and Nigeria), with no restrictions imposed by the government.

**Table 8:** Shelf‐life requirements for sourcing scenarios in selected countries in Latin America, Africa, and Asia regions

		Sourcing scenarios			
		A: Straights are imported	B: Premix are imported	C: Import of bulk finished product	D: Import international branded product
Shelf‐life requirement for MMS	Africa				
	Ghana	75% of active material intact on arrival Required labels and artworks subjected for approval by FDA. English used for all instructions on labels and inserts.			
	Kenya	Min 75%	Min 75%	Min 75%	Min 75%
	Nigeria	The shelf life varies from product to product, but the minimum and most common is 2 years but may be up to 3 years.			
	South Africa	50%	As close to 100% as possible	As close to 100% as possible	As close to 100% as possible
	Latin America				
	Brazil	For drugstores, they do not receive products with less than 1 year of remaining shelf life. Usually, MMS has 24–36 months of shelf‐life.			
	Colombia	For drugstores & supermarkets, they do not receive products with less than 1 year of remaining shelf life.			
	Guatemala	There are no requirements on shelf‐life			
	Mexico	Customer requirements—As a general standard, 24 months of shelf life is a target; 36 months would be ideal. Shelf life verified with stability studies	Customer requirements—As a general standard, 24 months of shelf life is a target; 36 months would be ideal	Customer requirements—As a general standard, 24 months of shelf life is a target; 36 months would be ideal	Customer requirements—As a general standard, 24 months of shelf life is a target; 36 months would be ideal
	Peru	24–36 months	24 months	24–36 months	24–36 months
	Asia				
	Bangladesh	Buyer's shelf‐life requirement is 75%	N/A	N/A	N/A
	India	Minimum 60% of the total shelf life at the time of clearance.			
	Vietnam	Not stipulated but over 12 months generally required for clearance in customs			

*Note*. MMS = multiple micronutrient supplement;

N/A = not applicable

#### 3.6.2 Label regulations

We inquired about the mandatory and voluntary requirements for labels of MMS for all 12 countries. Product label regulations are important for consumer awareness. They communicate what the product is, the ingredients, and for whom the product is intended. Label regulations also reflect the broader regulatory environment, and more items on the label are indicative of highly structured food and drug law. MIC with low manufacturing capacity, high regulatory controls may inadvertently support global companies at the cost of local companies because the former have the capacity to meet stringent requirements.

We found that all countries require at least the manufacture and expiry date and list of ingredients, as drug facts or nutrient facts, depending on how the product is registered in each country (see Table 5). Ingredient claims are voluntary across all countries and regions. Government endorsement on label is not allowed in Asia, Africa, or Peru. Health claims are allowed to appear on the labels of MMS sold in Guatemala, Peru, Bangladesh, India, and Vietnam. In Mexico and Colombia, health claims were not permitted on food supplements. In Ghana, Kenya, Nigeria, and South Africa, no disease reducing claims could be made, but ingredient and content claims are mandatory.

### 3.7 Local manufacturing capacity

Each of the countries surveyed indicated presence of local manufacturing capabilities of MMS. Local manufacturing capacity is defined as capacity of production facilities established in the country either by a multinational (mostly companies from the United States or Europe), regional (on the same continent), or a native company. These production facilities either purchase straights (Scenario A) or premix blends (Scenario B) to manufacture MMS tablets or capsules. Factory production cost for MMS by countries or regions are specified in Table 9.

**Table 9:** Range in factory production cost (USD) of multiple micronutrient supplement in selected low‐, middle‐, and high‐income countries^a^

Country or region	Cost per 30 capsules/tablets	Cost per capsule or tablet
Bangladesh	0.90–2.00	0.03–0.67
India	0.47	0.016
Vietnam	0.14–0.28	0.005–0.01
Japan	0.24	0.008
Latin America (Brazil, Colombia, Guatemala, Mexico, and Peru)	0.20–0.52	0.0067–0.017
United States	0.30	0.01

a Range in factory production cost (lowest–highest), as reported by key informants. For Latin America, similar ranges were reported for all countries. Factory production costs excludes packaging and sourcing of materials.

#### 3.7.1 Latin America

Overall in the LATAM region, retail sales of MMS grew by a cumulative annual growth rate of 14% from 2008 to 2013. In Brazil, Colombia, Guatemala, Mexico, and Peru, 197 million MMS were sold in 2014 by 13 local manufacturers (Strobel, 2014). Except for Mexico, each of the native companies had at least one leading brand. In Mexico, imported brands seem to be preferred. Most of the brands sold in LATAM are native companies except for those in Guatemala and one company in Colombia, where the production facilities were established by either a multinational or regional company. Ten companies import straights, with capabilities for blending vitamins and minerals, tableting or capsuling, and packing. All of the brands were produced in facilities that are WHO‐GMP certified. Total cost of production of MMS is highest in Brazil due to high import taxes on supplements. Mexico and Colombia are seen to be agile and competitive in manufacturing MMS. Except for Peru, all local manufacturers indicated an underutilized capacity and could increase production should there be a rise in demand for MMS.

In comparison, the Unites States has sales, on an average, of 200 million capsules or tablets of MMS each in the year 2014. It has several local manufacturers, and the cost of production in the United States is comparable to that in LATAM at USD 0.01 or less for a tablet or approximately USD 0.30 for 30 tablets or capsules (Table 9). However, the cost of production should be evaluated along with the quality of production because it affects the potency and performance of the MMS. We did not find any objective data on whether the quality of production in LATAM is comparable to that in the United States.

#### 3.7.2 Africa

There is local manufacturing capacity in Ghana for MMS in tablet form. All MMS brands are imported from the United Kingdom. Information for local manufacturing capacity in Nigeria was not available, despite being able to find one local brand. In Kenya, five WHO‐GMP certified manufacturers produce MMS. Four of them import ingredients indicating the existence of premixing and tableting or capsuling capabilities in Kenya. These five manufacturing facilities are stated to be underutilized and have the capabilities to expand. For very large scale mass production, capital goods (e.g., machine or equipment) would need to be imported. South Africa also has local manufacturers with premixing and tableting or capsuling capabilities that are underutilized. The largest production facility in South Africa is a multinational, which produces 3 million tablets of a leading brand of MMS for pregnancy.

#### 3.7.3 Asia

Overall in the Asia Pacific region, retail sales of MMS grew by a cumulative annual growth rate of 12% from 2008 to 2013. In Bangladesh, India, and Vietnam, there are 15 local manufacturers of MMS that produce 14 leading brands. Among these 15 local manufacturers, only two were multinational companies, one in India and one in Vietnam, which sold 5.5 and 30 million MMS, respectively, in 2014. The rest were all native companies. In 2014, nearly 110 million MMS were sold by the five native companies in Bangladesh and 133 million MMS by five local manufacturers in Vietnam. Five Indian local manufacturers have a cumulative installed capacity of 1.2 to 1.8 billion units (tablets or capsules) per year, whereas each manufacturer in Vietnam has an installed capacity between 3 and 7 billion units per year. Vietnam offers the most competitive factory production costs in this region (Table 9). However, we did not find data that compared the quality of finished products among countries in the region.

Indian and Bangladeshi manufacturers procure straights and those in Vietnam import premix. This indicates that tableting and capsuling capabilities exist in all the three countries, with India and Bangladesh also possessing blending capabilities. All countries in this region have WHO‐GMP certified facilities, with capacity to increase production if required. Both India and Vietnam have local equipment manufacturers to build new MMS manufacturing facilities or increase production capacity should there be a surge in demand, whereas Bangladesh would have to import equipment.

### 3.8 QA and QC

Although MIC expressed several challenges along the MMS or IFA value chains, no major barriers were expressed in HIC (i.e., Japan, United States and Germany) as long as actors along the value chain invested in high‐quality ingredients, infrastructure to maintain requirements for temperature and humidity, and a skilled workforce.

#### 3.8.1 Latin America

In Brazil, Colombia, Guatemala, Mexico, and Peru, high quality and consistent supply of ingredients (e.g., folic acid) was cited as a major barrier for sourcing. Ensuring homogeneity during the premixing or blending process was considered to be a challenge and hence requires investment in skilled workforce to ensure good manufacturing practices are being followed. There was not enough in‐country information on QC during transportation and in warehouses. Key informants reported that lack of capacity or knowledge for conducting quality checks on finished MMS is a major challenge for Colombia and Guatemala.

#### 3.8.2 Africa

Challenges along the MMS value chain are the same for all the four countries surveyed (i.e., Ghana, Kenya, Nigeria, and South Africa), and these challenges are also applicable to IFA supplements. Cost and time required for QA and QC processes were cited as key factors to consider for implementation. High costs are associated with the following: (a) hiring skilled workforce (or training) for the premixing or blending of ingredients and production of capsules or tablets; (b) laboratory controlled stability trials for subtropical or Mediterranean climate; and (c) materials for foil and container packaging, and road freight. Time delays are expected in sourcing high‐quality ingredients of pharmaceutical grade due to the cumbersome import process, and ensuring shipping and warehousing conditions are conducive for supplements. Further, adequate storage space for supplements in dark spaces below 25 °C in rural clinics and hospitals would be necessary.

#### 3.8.3 Asia

Two of the MMS manufacturers in Bangladesh recently obtained FDA approvals to export to the United States. Local manufacturers in Bangladesh meet almost all of the local demand and expressed minimal challenges along the value chain. Similar to other regions, homogeneity of the premix during the blending process is a major challenge in India and Vietnam. Concerns were expressed in sourcing poor quality ingredients in both countries. A specific concern was expressed in Vietnam that quality is often compromised in an effort to offer a low competitive price. Similar to other regions, maintaining adequate environmental conditions during transport, warehousing and storage will be a barrier for ensuring high‐quality MMS in this region.

## 4 DISCUSSION

This situation analysis has described the key elements of the market, manufacturing, and policies, which are likely to influence the local production of MMS in 12 MIC. We found significant variability across and within regions for local and sustainable production of MMS. Although there was manufacturing capacity and WHO‐GMP certification, with some countries even having local leading brands, not all countries are in a favourable position to produce locally. For example, in Ghana and Nigeria, local production of MMS will be more difficult to implement because of a small consumer market that prefers imported branded products. Local companies wanting to compete with international brands will have to invest significantly in marketing. Trade agreements and technology transfer could be leveraged to support affordable supply of MMS. Countries such as India and Vietnam, or HIC such as the United States, Germany, and Japan, could support MIC countries to improve their local manufacturing capacity through technology transfers, such as licensing agreements or joint ventures.

Guidelines and government oversight will need to be strengthened if governments consider switching from IFA to MMS. Comparison of 48 MMS for pregnancy shows that the manufacturers of these supplements in all the countries surveyed are not aware of the UNIMMAP formulation, which should be considered the benchmark formulation for antenatal MMS. Not one country had an MMS formulation that matched UNIMMAP. Several MMS in Category I had other essential micronutrients than those contained in UNIMMAP such as calcium, biotin, pantothenic acid, or DHA. Guidelines for the recommended formulation followed by dissemination through in‐country workshops with key actors are necessary to facilitate any transition from IFA to MMS.

Our survey point to gaps in technical capacity and QC practices in most of the MIC surveyed. There are several ways to improve QC and QA along the value chain. For example, countries could mandate better specifications and stability tests of both ingredients and final product. The stability test requirements should reflect the temperature and humidity conditions prevalent in the given country. In addition, ensuring shelf‐life requirements that exceed 18 months can improve quality from sourcing to point of purchase and home use. Finally, monitoring and surveillance systems need to be strengthened, with local food and drug authorities having better oversight on the MMS quality and potency. In some countries, this may require investments, either from private firms or governments, on technical capacity and laboratory equipment to test finished products for label claims (LeDoux et al., 2015). A guiding dossier and a resource plan to build capacity with food supplement or drug monitoring agencies for QC and QA throughout the supply chain would be required to ensure a safe transition from IFA supplementation to MMS. Such document has recently been created to support local manufacturing of micronutrient powders (United Nations Industrial Development Organization [UNIDO] and Home Fortification Technical Advisory Group [HF‐TAG], 2014).

Finally, this research also highlights the need for policy coherence along the entire value chain for MMS, from import to point of purchase, because various factors will influence the sustainability and affordability of local production of MMS. National governments may need to consider downstream (i.e., consumer‐facing policies) as well as upstream policies (i.e., manufacturing friendly policies) to achieve policy coherence.

Downstream policies include subsidy structures (e.g., vouchers) for purchases through market channels, value‐added tax exemption at point of purchase, or free access through clinics or hospitals at prenatal care, such that distribution and product cost is wholly absorbed by governments. Even if MMS is affordable and available, other barriers such as low compliance with antenatal care, poor health worker performance, poor counselling about product use and potential side effects (Galloway & McGuire, 1994), weak supply chain management systems (Victora et al., 2012), and consumer purchase capacity will continue to limit uptake (UNICEF, WHO and UNU, 1999).

Upstream policies to improve affordability include setting competitive and sustainable price ceilings coupled to import subsidies, import tax exemptions, commercial tax exemptions, company tax subsidies, or a combination of these. Taxes borne by the manufacturer create an incentive structure that makes MMS a premium product. In Colombia and India, for example, our data show that some nutritional ingredients, such as omega‐3 fatty acids, which have been shown to be beneficial during pregnancy for reducing risk on maternal depression and poor fetal neurodevelopment (Su et al., 2008), are subject to much higher import taxes compared to vitamins. Even in countries with strong local manufacturing capabilities, an affordable retail or wholesale selling price may depend on how governments structure import tax for straights, premix, and bulk products, as the manufacture is more likely to pass on the tax cost to the consumer (Food and Agriculture Organization, 2010).

Although this study provides a comprehensive overview of the manufacturing and policy factors, further in‐country examination will be required to improve the robustness of the data because we did not (a) assess the quality of products on sold in the market (USDA, 2015), (b) verify our key informant responses with national regulatory bodies, or (c) use representative sample of products. Furthermore, despite numerous follow‐ups with key informants, we still received incomplete data for some countries, namely, Germany and the United States. Missing data were common for market information, installed capacity, and QA/QC along the value chain. We mitigated these missing data points by triangulating with other data sources, such as IMD database and other key informants within DSM, and additional cross‐check by UNICEF on the regulatory data. Despite the limitations, we believe this to be an adequate representation of the key market and policy factors that are shaping the MMS availability in the 12 MIC countries that we sampled.

In contrast to MMS, WHO does recommend daily IFA supplement during pregnancy for reducing the risk of low birth weight and maternal anaemia or iron deficiency. There is nonetheless a significant lack of information on supply chains for IFA (Sununtnasuk, D'Agostino, & Fiedler, 2016). Although we did not specifically collect information on the production and procurement of IFA, a number of our informants reported problems for sourcing (raw material quality and blending), production (good manufacturing practices and QC), distribution (adequate storage facilities), and monitoring (shelf life). Therefore a number of these challenges would apply to both IFA (WHO 2016, UNIDO 2014) and MMS, although a situation analysis of IFA supply chain would be needed to estimate the factors that might impair the implementation of IFA. In one study that looked at the bottlenecks in the IFA supply chain in Senegal, stock outs at local level were common due to poor ordering processes and lack of funds for timely orders (Gueye, Pendame, Ndiaye, Diop, & Daff, 2015). Taken together, our data and the literature seem to suggest that procuring MMS in low‐income countries is likely to be more challenging than what is reported here for lower‐middle income countries.

In conclusion, this situation analysis offers a framework on the manufacturing and policy enablers and barriers for MMS local production. Importantly, a potential scale up from iron‐folate to MMS will need to be viewed holistically considering manufacturing, policy, and human capacity/skill factors, as these together will influence the implementation, sustainability, and affordability of MMS in MIC.

## CONFLICT OF INTEREST STATEMENT: DISCLAIMER AND DECLARATIONS OF INTERESTS

Sight and Life is the humanitarian, nutrition think‐tank, supported by DSM, which is a global life science company that produces nutritional ingredients used in micronutrient supplements. KGvZ is an independent consultant contracted by Sight and Life. GS is an employee of DSM.

RK and AF are UNICEF staff members. The opinions and statements in this article are those of the authors and may not reflect official UNICEF policies.

This manuscript was presented at the technical consultation “Multiple micronutrient supplements in pregnancy: implementation considerations for successful integration into existing of programmes” organized by the World Health Organization in collaboration with the United Nations Children's Fund and the Micronutrient Initiative (18–20 August 2015, Geneva, Switzerland). This paper is being published individually but will be consolidated with other manuscripts as a special supplement in Maternal and Child Nutrition, the coordinators of which were Gerardo Zamora, Roland Kupka, and Luz Maria De‐Regil. The authors alone are responsible for the views expressed in this paper; they do not necessarily represent the views, decisions, or policies of the institutions with which they are affiliated or the decisions, policies, or views of the World Health Organization. The opinions expressed in this publication are those of the authors and are not attributable to the conveners.

## CONTRIBUTIONS

ECM, KB, and KK conceptualized the study design and questionnaire. ECM and KB organized the data collection and led the analysis and interpretation of findings and drafting of the manuscript. KGvZ assisted in data organization and drafting of manuscript. KK, GS, RK, and AF helped in interpretation of findings and contributed to the critical revision of the manuscript for final submission. All authors approved the draft.
